# Data integration from pathology slides for quantitative imaging of multiple cell types within the tumor immune cell infiltrate

**DOI:** 10.1186/s13000-017-0658-8

**Published:** 2017-09-18

**Authors:** Zhaoxuan Ma, Stephen L. Shiao, Emi J. Yoshida, Steven Swartwood, Fangjin Huang, Michael E. Doche, Alice P. Chung, Beatrice S. Knudsen, Arkadiusz Gertych

**Affiliations:** 1 Department of Pathology and Laboratory Medicine, Cedars-Sinai Medical Center, Los Angeles, CA USA; 2 Department of Radiation Oncology, Cedars-Sinai Medical Center, Los Angeles, CA USA; 3 Department of Biomedical Sciences, Cedars-Sinai Medical Center, Los Angeles, CA USA; 4 Department of Surgery, Cedars-Sinai Medical Center, Los Angeles, CA USA

**Keywords:** Quantitative imaging, Tumor immune infiltrate, Immunohistochemistry, Image analysis, Breast cancer

## Abstract

**Background:**

Immune cell infiltrates (ICI) of tumors are scored by pathologists around tumor glands. To obtain a better understanding of the immune infiltrate, individual immune cell types, their activation states and location relative to tumor cells need to be determined. This process requires precise identification of the tumor area and enumeration of immune cell subtypes separately in the stroma and inside tumor nests. Such measurements can be accomplished by a multiplex format using immunohistochemistry (IHC).

**Method:**

We developed a pipeline that combines immunohistochemistry (IHC) and digital image analysis. One slide was stained with pan-cytokeratin and CD45 and the other slide with CD8, CD4 and CD68. The tumor mask generated through pan-cytokeratin staining was transferred from one slide to the other using affine image co-registration. Bland-Altman plots and Pearson correlation were used to investigate differences between densities and counts of immune cell underneath the transferred versus manually annotated tumor masks. One-way ANOVA was used to compare the mask transfer error for tissues with solid and glandular tumor architecture.

**Results:**

The overlap between manual and transferred tumor masks ranged from 20%–90% across all cases. The error of transferring the mask was 2- to 4-fold greater in tumor regions with glandular compared to solid growth pattern (*p* < 10^−6^). Analyzing data from a single slide, the Pearson correlation coefficients of cell type densities outside and inside tumor regions were highest for CD4 + T-cells (*r* = 0.8), CD8 + T-cells (*r* = 0.68) or CD68+ macrophages (*r* = 0.79). The correlation coefficient for CD45+ T- and B-cells was only 0.45. The transfer of the mask generated an error in the measurement of intra- and extra- tumoral CD68+, CD8+ or CD4+ counts (*p* < 10^−10^).

**Conclusions:**

In summary, we developed a general method to integrate data from IHC stained slides into a single dataset. Because of the transfer error between slides, we recommend applying the antibody for demarcation of the tumor on the same slide as the ICI antibodies.

**Electronic supplementary material:**

The online version of this article (10.1186/s13000-017-0658-8) contains supplementary material, which is available to authorized users.

## Background

In triple-negative and HER2+ early breast cancer, the presence of tumor-infiltrating lymphocytes (TILs) predicts for better response to neoadjuvant chemotherapy and improved overall survival [1–7]. In addition, the presence of TILs is associated with improved response to neoadjuvant Trastuzumab, a HER2-directed antibody whose efficacy depends in part on antibody-mediated cytotoxicity [3, 4]. In these studies, TILs are quantified according to the 2014 guidelines established by the International TILs Working Groups [8, 9]. Though the presence of TILs were associated with better outcomes, the optimal threshold of TILs in pretreatment core biopsies that predict a complete tumor response to Trastuzumab has not been established. One study reports that tumors in which ≥30% of the stromal area was occupied by TILs have more frequent complete pathologic responses to neoadjuvant Trastuzumab [10], while other studies found cut-offs as high as 60% [1]. The problem of the TIL cut-off maybe due to semi-quantitative scoring by pathologists, which is notoriously difficult to standardize and associated with significant intra- and inter-observer variabilities [9]. Digital image analysis has the potential to improve the accuracy and reproducibility of quantifying TILs. However, software for TIL measurements is not commercially available and will require careful validation against pathologists prior to its clinical deployment.

TILs include all mononuclear cells derived from the lymphoid, myeloid and granulocytic lineages such as lymphocytes (T cells, B cells, plasma cells, NK cells), granulocytes (neutrophils, eosinophils, basophils), monocyte/macrophages and dendritic cells. The anti-tumoral immune response is regulated by interactions between immune cell types [11]. Subpopulations of immune cells, specifically NK cells and certain T-cell subtypes, exhibit direct, cell-contact mediated cytotoxic activity against the tumor while other immune cells exhibit indirect anti-tumor activity through the expression of cytokines, chemokines and pro-apoptotic molecules. Therefore, to obtain a better understanding of the anti-tumoral activity by the immune infiltrate, individual immune cell types, their activation states and location relative to the tumor cells need to be quantified, in addition to the magnitude of the immune infiltrate as a whole. This requires enumerations of the immune cell subtypes separately in the stroma and inside tumor nests, which requires precise identification of the tumor area on a slide. To determine the composition of the immune cell infiltrate and its spatial relationship to the tumor and surrounding stroma, immunohistochemistry (IHC) with multiple antibodies is needed. Antibodies can be used either individually on slides, or they can be combined in a multiplex format. The number of slides impacts the analysis scheme of the tumor immune infiltrate and if multiple slides are used, the data must be combined into a single dataset prior to analysis. This dataset can then be used to calculate ratios between cell types [12, 13].

State-of-the-art imaging and quantification of cell populations in digital slides is accomplished through different instruments. One is the whole slide imaging instrument, such as the Aperio slide scanner, which is equipped with a RGB (red, green and blue channel) camera. The accompanying software allows quantification of up to 2 chromophores that are linked to the antibodies, in addition to the hematoxylin counterstain that labels all nuclei. The Aperio slide scanner is easy to operate and can be incorporated into a clinical workflow. The other instruments are for analysis of tissues in research projects. They include the Vectra-II imaging microscope, which utilizes a multispectral imaging approach that images large sections of slides coupled with software for color unmixing. This technology enables quantification of tissues stained with up to 6 antibodies. It employs the commercial OPAL staining system and inForm® software for staining and color unmixing and has proven to be a powerful approach in the quantification of up to 5 immune cell types in addition to demarcating the tumor [14]. Commercial software for analysis of the immune infiltrates include the Genie Spectrum (Aperio ePathology Solutions, Vista, CA) [15, 16], Image-Pro Plus 3.0 (Media Cybernetics; Silver Spring, MD) [17], Halo (Indica Labs, Corrales, NM) [18], ImageJ [19], and VMscope (VMscope GmbH, Berlin, Germany) slide explorer [9]. However, these software tools can only be used with slides stained with 2 antibodies. For samples with >2 target stains, the immune cell quantification may be carried out with the inForm® software that is provided with the Vectra-II multispectral instrument [20–22].

We developed a combined IHC and image analysis workflow to quantitate the immune infiltrate using 5 antibodies on 2 slides. Employing 2-plex and 3-plex chromogenic IHC protocols, we applied the workflow to a cohort of 81 HER2+ breast cancer patients and solved the following technical challenges: 1) computer-assisted, automatic transfer of the tumor mask outlines between slides, 2) generation of an analysis pipeline to quantify distinct immune cell types inside the tumor, on the tumor border and outside of the tumor, 3) determination of sources of error in association with the transfer of the tumor mask and affecting the regional quantification of immune cell populations.

## Methods

### Patient cohort

The study consisted of 81 consecutive cases of HER2+ breast cancer and was approved in IRB protocol #Pro00032113 at Cedars-Sinai Medical Center. Patients with Stage I-III HER2+ breast cancer who underwent surgery followed by chemotherapy and Trastuzumab from January 2005 through December 2011 were identified from the Cedars-Sinai Medical Center Cancer Registry. Patients who presented with Stage IV disease, whose tumor tissue was not available for marker evaluation, who did not receive follow-up at Cedars-Sinai Medical Center, and who did not receive chemotherapy and Trastuzumab were excluded. HER2 expression levels were determined based on clinical guidelines [23].

### Immunohistochemical staining of the same tissue section with CD45 and pan-CK antibodies

The antibodies were used in the sequence of CD45 ➔ Pan-Cytokeratin (Pan-CK) for staining of the same tissue section. For CD45, heat-induced epitope retrieval occurred with Na/EDTA pH 8.0 for ~30 min @ 90 °C. Tissues were blocked with animal-free protein blocking buffer (Vector Laboratories cat. # SP-5030) for 15 min. To quench the endogenous peroxide, the tissue was treated with H_2_O_2_ for 12 min. The anti-CD45 (Ventana Pre-Dilute, cat. # 790–2505) was applied for ~30 min @ 37 °C. Thereafter, the EnVision + System – HRP labeled polymer goat anti-mouse secondary antibody (Dako cat. # K400011) was used for 20 min, followed by DAB (3,3′-diaminobenzidine, Vector Laboratories cat. # SK-4100) for 8 min.

**Fig. 1:**
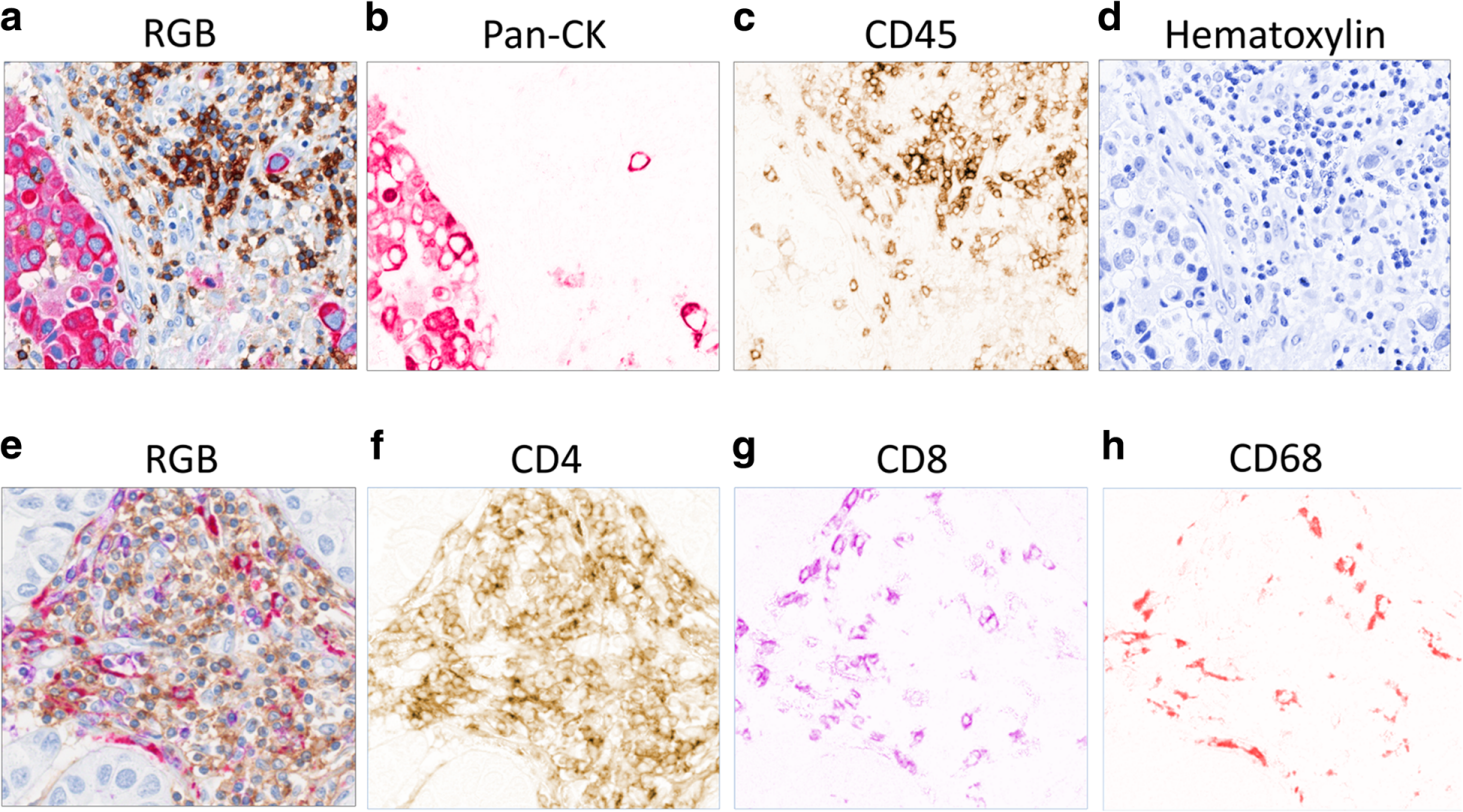
Visualizing individual antibody combinations before and after color separation in digital images. **(a-d)** Representative of invasive breast cancer stained with Pan-Cytokeratin (Pan-CK) and CD45. The tissue section was stained using an IHC protocol with 2 antibodies. The stained slide was imaged using the Aperio slide scanner and the colors were deconvoluted. **(a)** Original image of 2-antibody RGB stain, **(b)** Pan-CK+ breast cancer cells (red), **(c)** CD45+ lymphocytes (brown), and **(d)** hematoxylin positive nuclei (blue). **(e-h)** Representative image of tumor area parallel to **(a)** and stained with 3 antibodies**.** The stained slide was imaged using the Vectra-II slide scanner and the colors were unmixed. **(e)** Original RGB image of 3-antibody stain, **(f)** CD4+ cells (brown), **(g)** CD8+ lymphocytes (purple), and **(h)** CD68+ macrophages (red)

Next, slides were incubated with the denaturing buffer (citrate buffer pH 6) for 10 min @ 110 °C, to remove the CD45 antibody. Tissues were blocked with animal-free protein blocking buffer for 16 min. The Pan-CK antibody (Dako cat. # M3515) was diluted at 1:50 and incubated overnight @ 4 °C. The secondary antibody, ImPRESS anti-mouse alkaline phosphatase (Vector Laboratories, cat. # MP-5402), was incubated for 30 min. The chromogen VECTOR Red (Vector Laboratories, cat. # SK-5100) was applied for 10 min. Slides were stained with Modified Mayer’s Hematoxylin (American MasterTech Scientific, cat. # HXMMPT) for 1.5 min and cover-slipped. A micrograph of a region of interest from a slide stained with CD45 and Pan-CK antibodies is shown in Fig. 1a.

### Immunohistochemical staining of the same tissue section with CD4, CD8 and CD68 antibodies

One section per patient was subjected to multiplex IHC on the DISCOVERY ULTRA automated slide-stainer (Ventana, Tucson, AZ). The antibodies were used in the sequence of CD4 ➔ CD8 ➔ CD68 for staining of the same tissue section. For CD4, the heat induced epitope retrieval occurred with Na/EDTA pH 8.0 for ~40 min at 90 °C. To quench the endogenous peroxide, the tissue was treated with H_2_O_2_ for 12 min. The anti-CD4 (Ventana Pre-Dilute, cat. # 790–4423) was applied for ~40 min at 37 °C. Thereafter, the EnVision + System – HRP labeled polymer goat anti-rabbit secondary antibody (Dako cat. # K400211) was used for 32 min, followed by DAB for 8 min. To quench the remaining active HRP, slides were treated with H_2_O_2_ again for 24 min.

The heat-induced epitope retrieval for the next antibody, CD8, was repeated as described above. Tissues were treated with H_2_O_2_ for 12 min, followed by anti-CD8 (Ventana Pre-Dilute, cat. # 790–4460) for ~32 min at 37 °C. The EnVision + System – HRP labeled polymer goat anti-rabbit secondary antibody was incubated for 32 min. DISCOVERY Purple (Ventana, cat. # 253–4857) was applied as the chromogen to visualize CD8-antibody binding for 24 min.

Next, slides were incubated with the denaturing buffer (citrate buffer pH 6) for 10 min at 110 °C, to remove the CD8 antibody. Tissues were blocked with animal-free protein blocking buffer for 15 min. The CD68 antibody (Dako cat. # M0876) was diluted at 1:750 and incubated overnight at 4 °C. The secondary antibody, ImPRESS anti-mouse alkaline phosphatase, was incubated for 30 min. The chromogen VECTOR Red was applied for 10 min. Slides were counterstained with modified Mayer’s Hematoxylin for 1.5 min and cover-slipped. A micrograph of a region of interest from a slide stained with CD4, CD8 and CD68 antibodies is shown in Fig. 1e.

### Image analysis pipeline

**Fig. 2:**
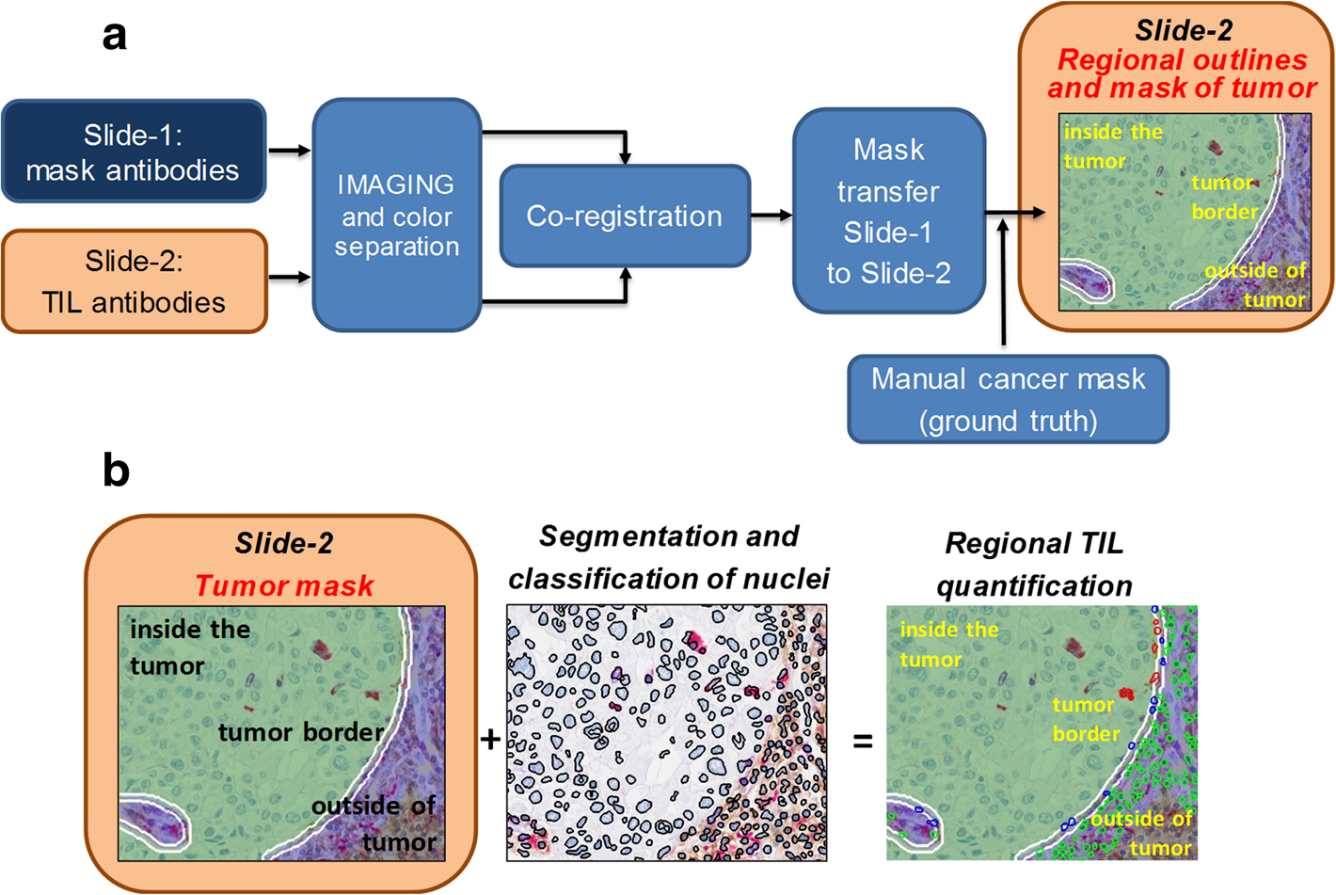
Image analysis workflow. **(a)** Workflow for regional separation. Corresponding ROIs within areas of invasive breast cancer in slide-1 and slide-2 are imaged and the colors are separated. The Pan-CK image is used to identify the cancer region in slide-1. Thereafter, cancer regions in slide-1 and slide-2 are co-registered based on the best overlap of the hematoxylin-stained nuclei. The mask is then transferred from slide-1 to slide-2. Alternatively, the cancer area in slide-2 is directly delineated by hand to generate the ground truth. **(b)** Workflow for TIL quantification. Nuclei are segmented in the whole image. Next, TILs are identified based on the color in the cytoplasm and counted within the tumor, at the tumor border and outside the tumor

We developed an image analysis workflow (Fig. 2) that employs one slide stained with Pan-CK and CD45 and a slide stained with TIL antibodies (CD8, CD4 and CD68) to quantify distinct immune cell types in regional areas of tumor (Fig. 2a). The workflow involves transferring of tumor mask from the Pan-CK slide to the slide with TIL makers by image registration. The performance of the mask transfer is evaluated against manual ground truth tumor delineations on the slide with TIL markers, and by counting of TILs in regional outlines of the tumor. This pipeline is also used to correlate counts of the TIL constituents inside and outside of the tumor (Fig. 2b).

### Imaging of slides and identifying high-density cancer areas

Slides stained with Pan-CK were imaged using a high-resolution whole-slide RGB scanner Aperio AT Turbo with a 20× objective (Leica Biosystems, Vista, CA). To detect regions of interest (ROI) with a high-density of cancer cells, the color-deconvoluted whole slides of Pan-CK images were thresholded to provide an epithelial mask. Only areas of invasive breast carcinoma were analyzed, and image tiles of ductal carcinoma in-situ and normal glands were excluded from the analysis according to the guidelines provided by the International TILs Working Groups [7, 8]. Tumor nuclei detected under a Pan-CK mask were counted to obtain local cell density maps (counts/mm^2^). The counting was carried out on square image tiles that covered the entire tissue area of the slide. The cell density map was visualized in 3-D to identify ROIs of high tumor cell burden for subsequent analyses (Additional file 1: Fig. S1). Next, coordinates of the ROIs were transferred from the 3-D map to the slide stained with the triplex-IHC. These ROIs was then imaged on the multispectral tissue imaging platform Vectra-II (Perkin-Elmer, Waltham MA). The Vectra-II is equipped with a scientific-grade charge-coupled device monochromatic camera and a 20× objective. The exposure time was 4 ms, and a 1 × 1 pixel binning was used for image acquisition (Nuance, Perkin-Elmer). The spectral image cubes encompassed the cancer areas. Cubes were acquired in the spectral range between 420 nm and 700 nm with 5 nm resolution yielding 31 wavelength specific images per cube. Each image in the cube had 1040 × 1392 pixels (pixel size = 0.5 μm × 0.5 μm) in horizontal and vertical direction. A flat-field correction was applied to prepare the cube for digital unmixing of chromophore colors.

### Color deconvolution and spectral unmixing

Two approaches were used for color separation of images scanned with Aperio or Vectra-II slide scanning platforms (Fig. 2). For digital images obtained with the Aperio slide scanner, color deconvolution, a method to transform RGB color tissue images into individual images depicting the concentration of each chromophore, was applied to separate the brown (CD45) and red (Pan-CK) colors from blue (hematoxylin). Briefly, the absorbance values of chromophore mixtures are decomposed into absorbance values of single chromophores. RGB images of the mixture of chromophores in the stained tissue and RGB spectra of single chromophores are provided as respective input data. The fingerprints that specify the RGB components of single chromophores were respectively set for DAB = [0.268, 0.570, 0.776], FastRed = [0.214, 0.851, 0.478], and hematoxylin = [0.490, 0.769, 0.410] in the code color deconvolution ImageJ plugin [24] that was applied to isolate monochromatic images of FastRed (Pan-CK) for further analysis and identification of cancer areas. Antibodies visualized after color deconvoluted images are shown in Fig. 1b-d.

In the second approach, DAB, FastRed, V-purple and hematoxylin images were spectrally unmixed for cancer area image co-registration and cell quantification using the inForm® software of the Vectra-II. Briefly, spectral cubes of cancer areas were digitally unmixed to obtain monochromatic images representing optical density of the chromogens (Fig. 1f-h). Spectral fingerprints specific for individual chromogens were used to determine the proportion of the spectrum from each chromogen in the spectrum of mixed chromogens [25]. The spectral fingerprints were collected from separate slides that were stained with single primary antibodies and corresponding chromogens.

### Cancer cell mask

Spectrally unmixed Pan-CK images were exported from the Vectra-II and imported by into an image processing tool that we developed to obtain the cancer cell mask. The Pan-CK image was thresholded using an automated histogram thresholding procedure [26, 27]. The resulting binary image was post-processed. Small objects were removed by applying morphological image opening [28]. Morphological closing was subsequently applied to smoothen the cancer cell mask boundaries. The final mask was exported for the image co-registration procedure.

### Image co-registration

We implemented the affine image co-registration procedure [29] in order to transfer the cancer cell mask of the cancer area to the image of the immune infiltrate. The hematoxylin images from both slides were used in the co-registration procedure to calculate the co-registration transformation matrix [29]. The matrix contained a set of parameters to direct the transfer and alignment of the images. After co-registration, the contour of the transferred mask was manually adjusted to resolve discrepancies in tumor architecture between the two slides.

### Nuclear segmentation and classification of immune cells

Unmixed images were also imported to inForm 2.0 tissue image analysis software (Perkin-Elmer, Waltham MA) to detect and classify subtypes of immune cells. First, cell nuclei were detected based on the local concentration of hematoxylin and delineated by a nuclear segmentation algorithm (Fig. 2b). The circular nuclear outlines were subsequently expanded by a constant number of pixels which corresponded to the length of half of the mean radius of an immune cell nucleus. The space between the two concentric circles was defined as the cytoplasmic mask. This mask was overlaid respectively onto unmixed images of CD4 (brown), CD8 (purple) and CD68 (red) triple stains to classify these cells for enumeration. The average pixel intensity in the cytoplasmic mask was used to establish a threshold [26]. Cells positive for individual stains were counted and the number of negative cells under the co-registered cancer mask was used as the reference measurement (Fig. 2b).

### Regional outlines of tumor

After transferring the tumor mask, we established 3 regions to enumerate the immune infiltrate: 1) the intra-tumoral region as defined by the manually corrected mask, 2) the tumor border region – a ring along the tumor edge measuring 7 pixels (3.5 μm), and 3) the extra-tumoral region – a second ring to the tumor border region measuring 50 pixels (35 μm) (Fig. 2b).

### Cancer mask transfer evaluation, data analysis and visualization

Two parameters were measured to assess the performance of cancer mask transfer between slides: a) the overlap ratio (*Ov*) that represents the normalized number of pixels that are both under the transferred mask and under the ground truth cancer area (delineated by a pathologist) and defined as follows:

1 $$ Ov=\kern0.5em {\#}_{pixels}\left( CMSK\cap GT\right)/{\#}_{pixels} GT $$

where: *CMSK* is the co-registered binary cancer mask, and *GT* is the binary manual ground truth of cancer mask. ∩ is the logical intersection of the involved masks, and #_*pixels*_ is the pixel count. *Ov* reaches 1 for the perfect concordance (overlap) of *CMSK* and *GT*, and 0 if the masks do not overlap at all.

b) The tumor cell count error (*TCe*) that was measured as the relative difference between the number of tumor cells under the transferred cancer mask and the number of cells under the ground truth cancer mask, which was manually delineated by the pathologist. *TCe* is defined as follows:

2 $$ TCe=\kern0.5em \left|{\#}_{cells} CMSK-{\#}_{cells} GT\right|/{\#}_{cells} GT $$

where: the #_*cells*_ is the tumor cell count under the respective masks, and | | is the absolute value operator.

*TCe* reaches 0 in case the number of cells under the respective masks are the same, or 1 in case there are no tumor cells under the transferred mask.

Intra-tumoral, tumor border and extra-tumoral regions were demarcated in 358 ROIs from 81 cases. First, tumor cells were counted in the intra-tumoral region. Cells positive for CD4, CD8, CD68 and CD45 were counted in intra-, border- and extra-tumoral regions. Bland-Altman plots and one-sided *t*-test were used to investigate differences between immune cell counts underneath the manually annotated versus transferred tumor masks.

In order to investigate the effect of the tumor growth pattern on the mask transfer, three tissue blocks with breast tumors that contained areas of solid and glandular growth pattern were used to obtain consecutive re-cuts from each block. 10 re-cuts from each block were stained with Pan-CK and counterstained with hematoxylin. Areas displaying solid and glandular growth patterns were identified and imaged with the multispectral instrument. The Pan-CK stain was spectrally unmixed and used to build a cancer mask for each image. The overlap *Ov* between masks was calculated as a function of the distance between the re-cuts and the tumor growth pattern. One-way ANOVA was used to evaluate discrepancies in *Ov* rates derived from solid and glandular areas.

Image data analysis, performance evaluation, 3-D cell density map visualization and definition of regional outlines of tumor were coded in Matlab programming environment (The MathWorks, Natick, MA). Our previously developed tool for ground truth tissue annotations [30] was used here for the manual cancer mask editing and for generating the ground truth by the pathologist.

## Results

To quantify TILs within and surrounding nests of tumor cells in regions of invasive breast carcinoma, we conducted IHC staining with 5 antibodies in a cohort of 81 cases of HER2+ invasive breast carcinoma. Slide-1 was stained with 2 antibodies that outlined the cancer (Pan-CK) and lymphocytic immune infiltrate (CD45). Slide-2 was stained with 3 antibodies to separately identify CD4+ T-cells, CD8+ T-cells and CD68+ macrophages. After scanning on the Aperio AT Turbo, the digital image of slide-1 underwent color-deconvolution (Fig.1a-d). The red Pan-CK images were processed to identify areas with high densities of tumor cell nests through visualization in a 3-D cell density map of the entire slide. Based on this map, 3 to 5 regions of interest (ROI) were randomly selected per slide, totaling 358 ROIs for further analysis (Additional file 1: Fig. S1). Coordinates of the 5 ROIs were marked on slide-1 and transferred to slide-2 stained with the immune cell antibodies. The corresponding ROIs in slide-2 were imaged on the Vectra-II multi-spectral imaging platform (Fig. 1e-h). Several controls were performed to validate the antibody staining and to evaluate the IHC staining procedure. First, we only employed clinical grade antibodies that are normally used for patient care. Second, we tested the sensitivity of antibodies to up to 4 sequential heat retrieval steps. The staining intensities of antibodies in our panels were not affected by antigen retrieval (data not shown). Finally, we excluded the possibility of incomplete antibody removal during the heat retrieval treatment. Incomplete dissociation of antibodies causes reactivity with secondary antibodies and coloration by 2 or more chromophores. We did not observe cells that were stained with more than one color as demonstrated by comparing the panels in Fig. 1, suggesting that the antibodies were effectively denatured by the heat retrieval treatment. In contrast to the Aperio software, the Vectra-II/ inForm® software is best suited for the analysis of select ROIs. In addition, only inForm® can be used for color separation of more than 3 colors.

**Fig. 3:**
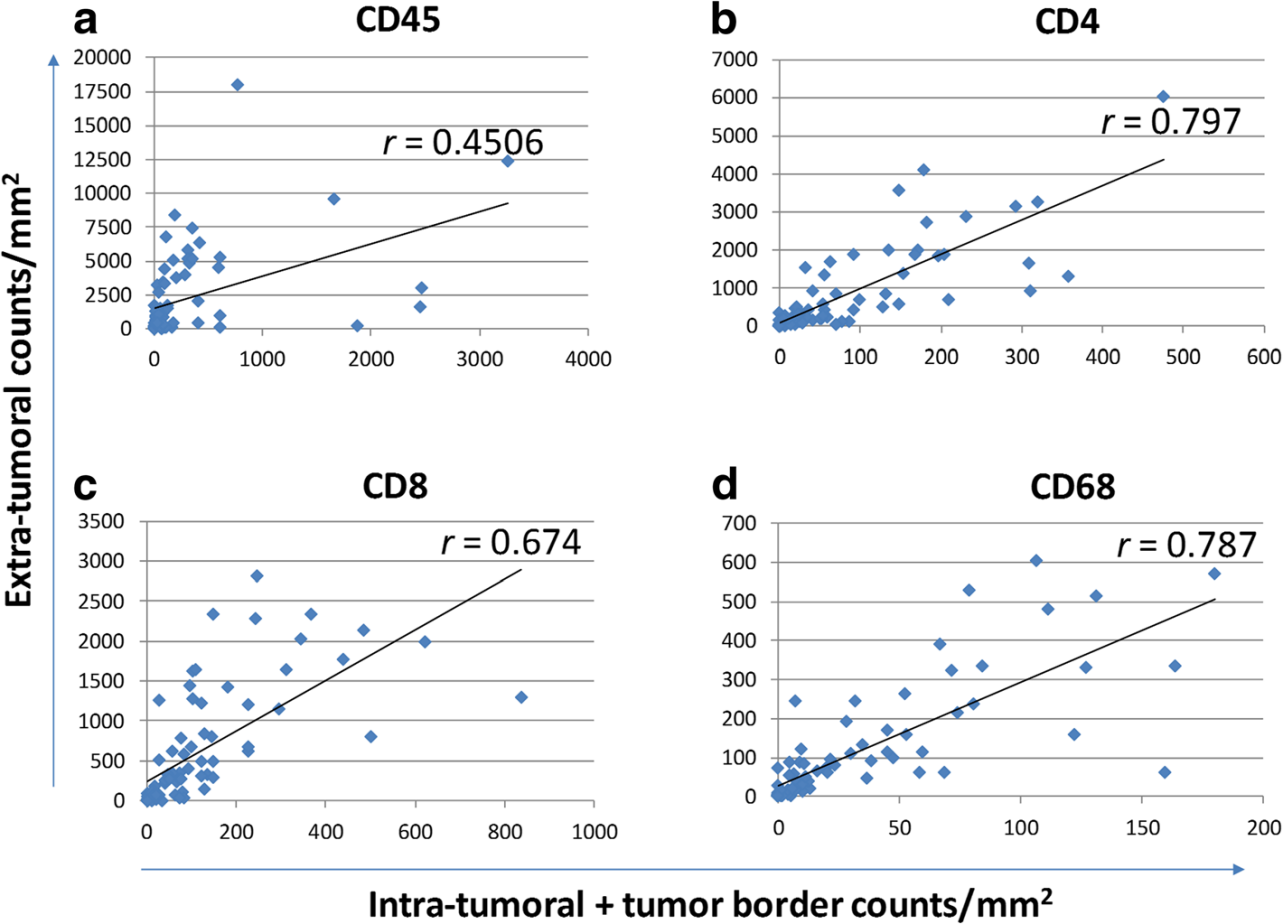
Correlation between intra- and extra-tumoral immune infiltrate. Tumor areas were outlined by hand in images from 81 cases. **(a)** CD45+ cells were counted in slides stained for CD45 and Pan-CK, after regional separation of intra-tumoral, tumor border and extra-tumoral regions. The y-axis represents cell counts per mm^2^ outside the tumor area, while the x-axis depicts cell counts within tumor nest plus cell counts at the border. The Pearson’s correlation coefficient is shown. **(b)** CD4+**, (c)** CD8+**,** and **(d)** CD68+ immune cell numbers were obtained from slides stained with the 3-antibody IHC protocol and analyzed as described in **(a)**. Pearson’s correlation coefficients are shown for concordance of TIL subtypes inside and outside nests of tumor cells

According to the recommendations of the International TILs Working Groups, only those mononuclear immune cells (primarily lymphocytes and macrophages) outside tumor cell glands or clusters in areas of invasive breast carcinoma were assessed. Further, the amount of TILs in the tumor microenvironment is reported as the area occupied by TILs divided by the total area of stroma. TILs within or at the border of tumor nests were excluded. The semi-quantitative assessment of TILs by pathologists generates an intra- and inter-observer variability that complicates establishing clinically relevant thresholds of TILs. Since digital quantification of TILs can solve this problem, we first established the pipeline of counting CD45+, CD4+, CD8+ and CD68+ TILs inside and outside tumor areas that were manually outlined (Fig. 2a). A moderate correlation between intra- and extra-tumoral CD45+ lymphocytes (T-cells and B-cells) with a Pearson correlation coefficient of *r* = 0.45 was observed. Pearson correlation coefficients were greater for CD4+ (*r* = 0.797), CD8+ (*r* = 0.674) and CD68+ (*r* = 0.787) immune cells (Fig. 3). These data suggest that for T-cells, the TIL numbers in the tumor microenvironment are associated with the intra-tumoral T-cell infiltrate. Furthermore, the lower correlation of CD45+ lymphocytes points to different distributions of T-cells and B-cells.

In order to increase the efficiency of outlining tumor regions, we replaced the time consuming hand annotation of tumor nests by a computer-assisted process. The tumor mask was outlined in slide-1 using a cytokeratin antibody IHC approach and transferred to slide-2, that contained the TIL antibody stain (Additional file 1: Fig. S2). For comparison, the tumor was manually outlined by a pathologist in slide-2. TIL numbers were obtained through both annotation methods (Fig. 2). To identify TILs, the first step consisted of a segmentation of all nuclei in the ROI. Next, cells were annotated based on the color outside the nuclear contour and classified as CD4+, CD8+ or CD68+. Finally, individual cells inside, at the border and outside the mask were counted (Fig. 2b).

To align the cancer masks in both slides, areas with high cancer cell densities (Additional file 1: Fig. S1) were co-registered based on the nuclear hematoxylin stain (Additional file 1: Fig. S2). The distortion parameters in the cancer mask in slide-1 that were required for co-registration with the cancer in slide-2 were described in the transformation matrix. After its transfer to slide-2, the cancer mask was further adjusted using the parameters in the transformation matrix to achieve the greatest overlap with the tumor area in slide-2. The final cancer mask in slide-2 was used as a starting point to define 3 cancer regions, the inside of the tumor, the tumor border and extra-tumoral region (Fig. 2b).

**Fig. 4:**
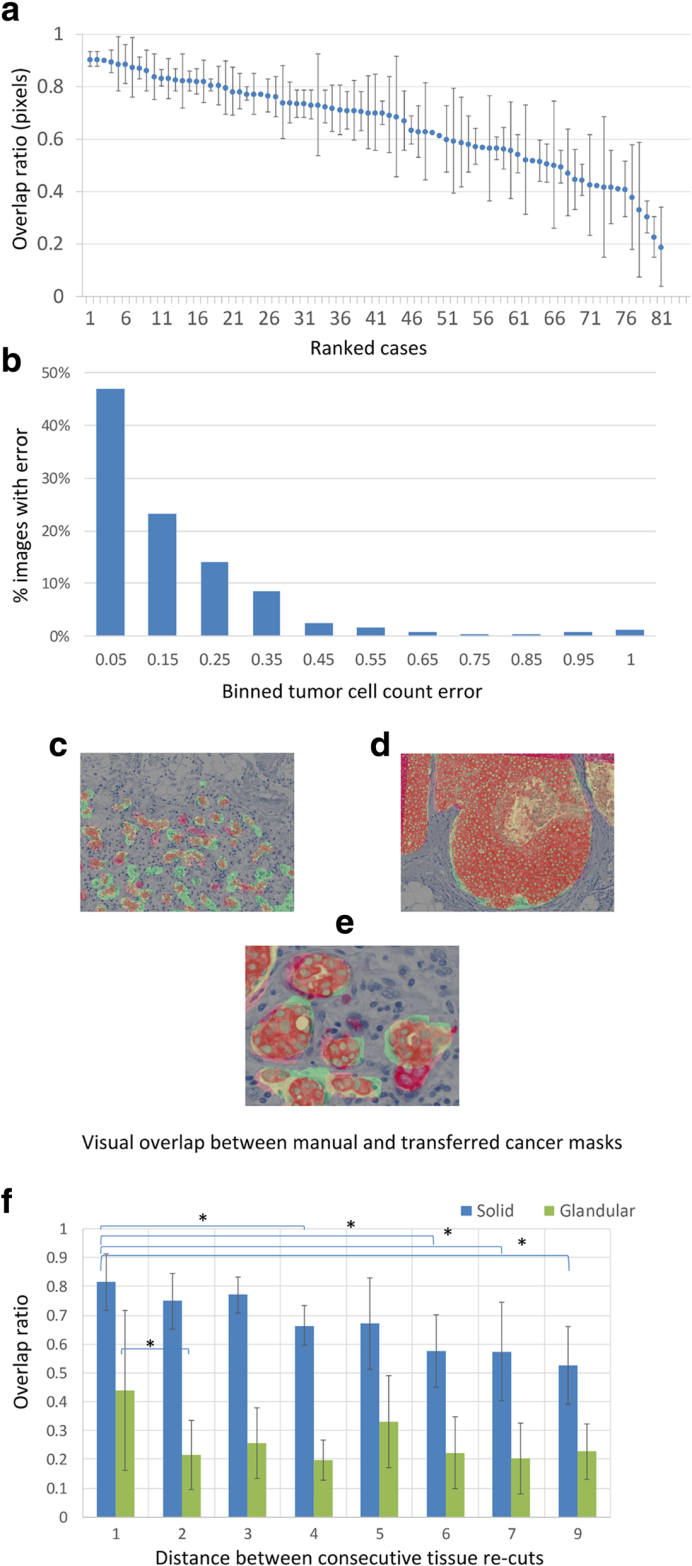
Overlap between manual and transferred cancer masks. **(a)** Error in alignment of transferred and ground truth masks. The overlap ratio was calculated as described in Materials and Method and plotted on the y-axis. The mean represents the average overlap in 3–5 images from each case and the standard deviation is indicated by the error bars. Cases are ranked in descending order of overlap ratio along the x-axis. **(b)** *Distribution of cell count error.* The difference of tumor cell counts underneath the transferred versus ground truth tumor masks was determined. 358 images were assigned to bins based on the error of tumor cell counts (see Materials and Methods) underneath the transferred tumor mask. **(c-e)** Visual demonstration of the error inflicted by the mask transfer. The transferred mask is outlined in green and the ground truth mask in red. Notice the association between the magnitude of error and the growth pattern by comparing the glandular growth pattern in **(c)** and **(e)** and solid growth pattern in **(d)**. **(e)** shows a close-up of an area in **(c)**. **(f)** Comparison of the error of mask transfer in solid (blue) versus glandular (green) regions of invasive breast cancer. Serial sections from 3 tumor blocks of invasive breast cancer were stained with Pan-CK. Solid and glandular areas were separately annotated and the masks transferred between slides. The x-axis indicates the distance between serial sections (unit length = 4 μm) from the same block and the y-axis demonstrates the overlap ratio of the transferred versus hand-annotated masks within solid versus glandular regions of invasive breast cancer. The difference in the overlap between solid and glandular regions was significant (* = *p* < 0.05)

To determine the accuracy of the mask transfer, the areas under the transferred hand-delineated (ground truth) cancer masks were compared. The accuracy of the mask transfer was determined by the ratio in pixels with the area of overlap between the masks divided by the pixels in the ground truth mask (see eq. 1 in Materials and Methods). Alternatively, cell counts were used instead of pixels (see eq. 2 in Materials and Methods). Figure 4 shows results of the error that arises from transferring the cancer mask in 81 cases. In Fig. 4a **,** cases were ranked based on increasing error, as indicated by the decrease in the overlap of the masks. The graph shows the mean and standard deviation of the overlap ratio of 3–5 ROIs from each case. The mean overlap ratio across 358 ROIs was 0.679 ± 0.18 and the median overlap ratio was 0.723. Next, the error caused by the mask transfer in terms of the tumor cell count was determined. Differences in tumor cell counts between the transferred and ground truth masks were normalized to the cell count underneath the ground truth mask and equaled zero for a perfect overlap between masks. The median of error in the tumor cell count was 0.109, which amounts to ±10.9% of the tumor cell count in the hand-annotated mask. Each ROI was assigned to a bin based on the error, and the distribution of the bins is presented in Fig. 4b.

In addition, we aimed to distinguish between 2 sources of error in the mask transfer, which are the error that is caused by the tumor growth pattern and the error that is caused by the distance between parallel re-cut slides. For this purpose, we selected 3 cases that had both solid and glandular tumor growth patterns. All slides were stained with Pan-CK and areas of solid and glandular tumor growth patterns were annotated. In addition, the distance between consecutively recut slides was recorded. Figure 4c demonstrates a glandular growth pattern, whereas Fig. 4d reveals a solid growth pattern. A close-up view of a sub-region in Fig. 4e illustrates the detailed overlap between the manual ground truth (red) and the transferred cancer mask (green). Overlap ratios for solid (blue bars) and glandular (green bars) tumor growth patterns are separately shown in Fig. 4f. The highest overlap ratios were observed for the solid growth pattern (0.814 ± 0.097) and lesser overlap ratios were noted for glandular growth patterns (0.438 ± 0.277). A one-way ANOVA revealed a significant difference in the overlap ratios between the aggregate of all solid versus glandular regions (*p* < 10^−6^). The first significant difference in the transfer of masks associated with solid growth patterns occurred after the 4th slide (*p* = 0.039), while for glandular growth patterns the transfer of the tumor mask to an adjacent slide was already associated with a significant difference in the overlap ratio.

**Fig. 5:**
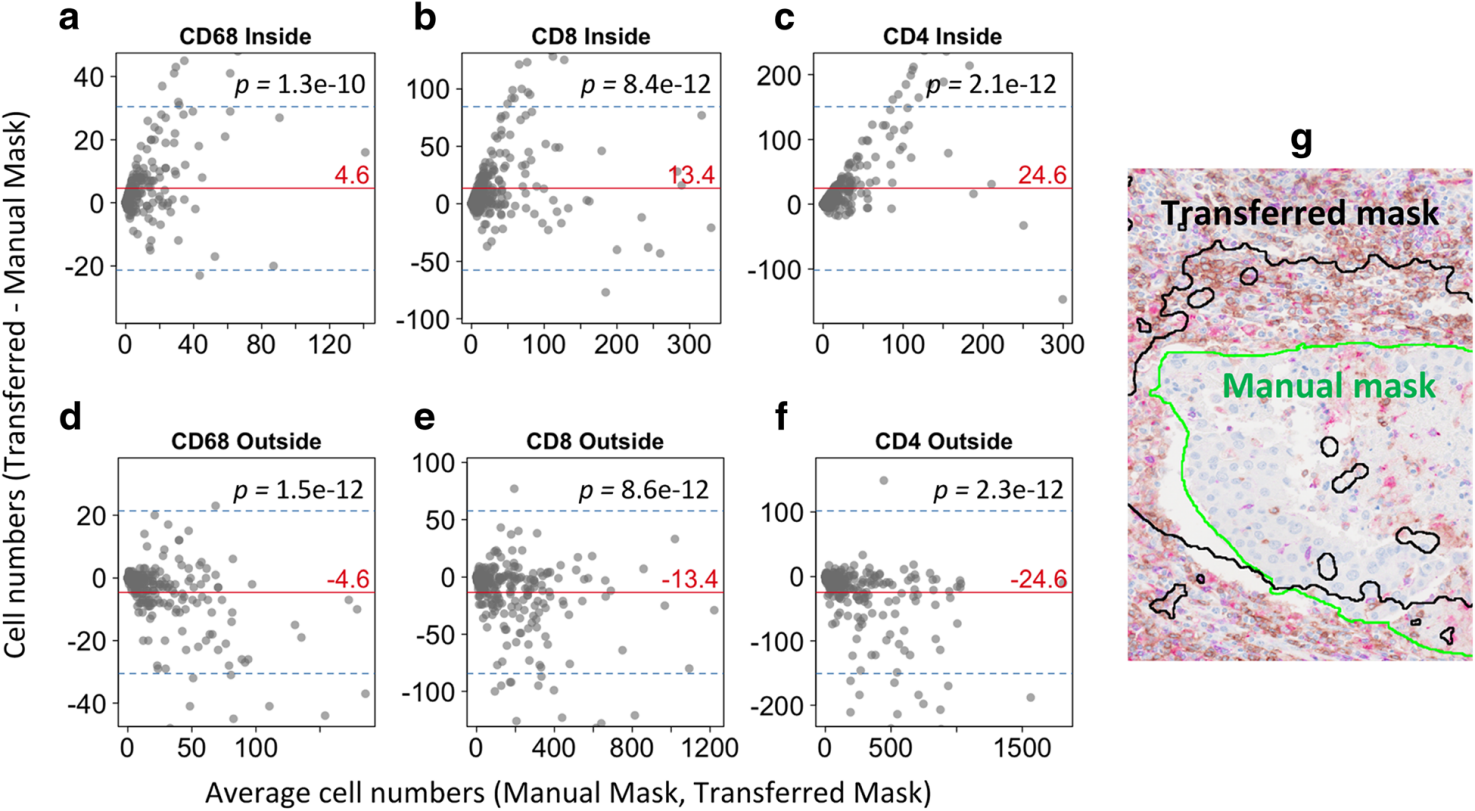
Error in immune cell counts caused by tumor mask transfer. Bland-Altman plots illustrating errors of immune cell subtypes inside and outside the tumor mask. **(a-c)** The difference of CD68+, CD8+ or CD4+ counts between transferred and ground truth tumor masks is plotted on the y-axis. The x-axis represents the mean cell count underneath transferred and manual masks. The red line indicates the mean error and blue lines the 95% confidence interval of the error. *p*-values were calculated by a one-sided *t*-test. Zero indicates perfect concordance in cell counts underneath both masks. **(d-f)** Same as **(a-c)** but for TIL numbers outside the tumor area. **(g)** Representative image of transferred versus ground truth tumor mask

Next, we questioned whether the error of the mask transfer affected the TIL counts inside and outside the tumor mask. TILs were counted in 81 slides underneath and outside of the transferred or hand-annotated tumor masks. When calculating the difference of cell counts underneath the transferred and hand-annotated masks and using Bland-Altman plots to visualize the data, error rates in TIL counts were high for all TIL subtypes (Fig. 5). A significant difference in TIL counts was observed using a one-sided *t*-test both inside and outside the tumor. This result demonstrates that the error of the mask transfer propagates to the enumeration of immune cells inside and outside the tumor mask.

## Discussion

The main goal of the study was to establish the quantitative method to profile the immune infiltrate of cancers and distinguish intra and extra-tumoral inflammation. A secondary goal was to dissect cell populations in the immune infiltrate that can be distinguished through IHC. We employed a combination of IHC with 5 antibodies with digital image analysis to demonstrate good concordance of intra- and extra-tumoral CD4+ CD8+, CD68+ cell numbers. Next, to improve the laborious annotation of tumor areas we established a pipeline for the transfer of tumor mask from one slide to another. The transfer required co-registration of slides based on hematoxylin stained nuclei. However, we observed a significant error in the mask transfer, dependent on the tumor growth pattern that led to differences in tumor and immune cell counts underneath the transferred mask compared to the ground truth. Altogether, we demonstrate for the first time the application of chromogenic staining method in order to visualize the composition of immune infiltrate with a regular brightfield microscope. Based on the error of the mask transfer that is caused by the biological variability between slides, combining data from sequential slides reduces data quality. Therefore, antibodies reactive with TILs or cancer cells should be used on the same slide.

A major limitation of our process is caused by the biological variability between tissue sections from the same tumor, resulting in an error that cannot be corrected by a simple approach. It is well known that protein expression varies between adjacent tissue sections of a tumor because of biological variability within tumor regions [31]. However, the degree to which the cellular composition and tissue architecture change between adjacent tissue sections remains poorly understood. Our recent study employing 7 antibodies on individual slides was compromised by errors arising from biological variability between slides [32]. During this study, we realized the value of multiplex IHC. Therefore, to overcome the problem of biological variability in subsequent studies, we developed a multiplex staining approach. The number of antibodies that can be applied sequentially in the chromogenic assay was shown to be limited to 3 [33]. Since staining with more than 3 antibodies compromised tissue quality we did not exceed a triple IHC stain. This, in turn, prompted the need for data integration from 2 slides to profile the composition and location of the tumor immune infiltrate. This approach allowed us to conduct TIL measurements related to the spatial organization of the tumor in whole slides.

Tissue micro arrays (TMAs) may also be useful in assessment of TILs, particularly for the rapid evaluation of large clinical cohorts [9]. Our method to quantitate the immune infiltrate could be in principle applied to tissues from TMAs. However, the use of TMAs comparing to re-cuts from tumors fixed on whole slides for TIL quantification would be limited for the following reasons: a) TMAs may not contain enough tissue to reliably determine the regional outlines of tumor, b) TMAs that contain only tumor cells or only stromal components would be unsuitable to determine correlations between intra- and extra-tumoral immune infiltrate, and c) since TILs generally form in the peritumoral regions, they may be absent or underrepresented in TMAs from heterogeneous tumors [9].

Recently, tissue staining with 5 to 6 antibodies on the same tissue section has become possible with commercial reagents, such as the OPAL kit from Perkin-Elmer or the DISCOVERY 5-plex procedure from Roche-Ventana. However, in contrast to our chromogenic approach, these detection systems employ fluorescent probes. A fluorescent analysis of CD4+, CD8+ and CD20+ lymphocytes in breast cancer using fluorescently tagged antibodies and the AQUA image analysis system demonstrated an association of AQUA scores and pathologic complete response in a cohort of patients treated with neoadjuvant chemotherapy [34]. More advanced systems allowing high-dimensional cytometric application include tissue CyTOF, which consists of a quadrupole –TOF. Droplets containing single, nebulized cells and bound high-metal conjugated antibodies are analyzed and the amount of bound antibody is related back to the tissue [35]. The limitations of fluorescent staining include the reduced durability of the stain, the difficulties with diagnostic evaluations of stained cells and problems with the analysis of digital, fluorescent slides by practicing pathologists. On the other hand, fluorescent probes have the advantage that they can be used with antibodies that react with proteins in the same subcellular compartment. To improve the diagnostic accuracy, it is possible to artificially colorize fluorescent signals and to establish the impression of a chromogenic IHC image.

The multiplexing of more than 3 antibodies with different chromogens for visualization with a regular microscope is currently not available as a commercial kit. The advantages of this type of multiplex IHC are its stability and ease of inspection of the whole slide with a regular microscope. The clinical utility in the short term is therefore greater using the chromogenic IHC method. However, the approach also has limitations. Staining with antibodies that bind to proteins in the same subcellular compartment cause problems with interference between antibodies due the reduced access of the second antibody by the chromogen precipitate from the first antibody. In addition, the analysis of slides requires a complicated process of color unmixing, the linear range of the chromogens is less than that of the fluorophores and the validation of the chromogenic assay is more complicated than validation of fluorescent tissue staining. However, chromogenic assays are clearly advantageous when utilizing antibodies that do not overlap in their subcellular staining patterns.

## Conclusions

We developed a novel chromogenic tissue staining and digital image analysis pipeline that permits the integration of data from 2 digital images and 5 antibodies. Using an automated transfer of the tumor mask between slides enabled the analysis of the immune infiltrate in tumor areas and enumeration of 3 immune cell types within the immune infiltrate. The error in the quantification of immune cells that was generated through the mask transfer was associated with the tumor growth pattern. Altogether, multiplex based IHC coupled with digital image analysis has the potential to improve the extraction of data related to the immune infiltrate of tumors.

## Additional file

Additional file 1:Fig. S1 Visualization of staining components used to generate a 3-D cell density map of cancer areas. **(a)** A whole-slide multicolor RGB bright-field image of breast cancer tissue stained for CD45 (brown), Pan-CK (red) and a hematoxylin nuclear counter-stain (blue). **(b-d)** The whole-slide multicolor RGB image was color-deconvoluted to separate the staining components: **(b)** CD45+ image, **(c)** Pan-CK+ image, and **(d)** hematoxylin image. **(e)** A 3-D cell density map of Pan-CK-positive cells (which demarcates areas of epithelial cells) was derived from the Pan-CK component image. Areas of benign glands and in-situ ductal carcinoma were visually excluded by a pathologist. A threshold applied to the map lead to the identification of cancer areas which were processed to segment out a cancer mask. **(f)** The locations of the cancer mask were transferred to a tissue image with no Pan-CK staining to enable the enumeration of immune cells in IHC stained slides. **Fig. S2** Transfer of cancer mask from slide-1 to slide-2. The cancer mask is generated in slide-1 based on Pan-CK staining. Hematoxylin-stained nuclei in slide-1 are identified and co-registered with corresponding, hematoxylin-stained nuclei in slide-2. A transformation matrix is established based on co-registered nuclei to transform the cancer mask in slide-1 for alignment with slide-2 in the whole ROI. After co-registration the cancer mask is transferred from slide-1 to slide-2. (PPTX 1673 kb)

## Abbreviations

ANOVA: Analysis of variance
CMSK: Co-registered binary cancer mask
GT: Binary manual ground truth of cancer mask
HER2: Human Epidermal growth factor Receptor 2
ICI: Immune cell infiltrates
IHC: immunohistochemistry
Ov: Overlap ratio
Pan-CK: Pan-Cytokeratin
RGB: Red, green, and blue channel
ROI: Region of interest
TCe: Tumor cell count error
TILs: Tumor-infiltrating lymphocytes

## References

[1] Denkert C, Loibl S, Noske A, Roller M, Muller BM, Komor M (2010). Tumor-associated lymphocytes as an independent predictor of response to neoadjuvant chemotherapy in breast cancer. J Clin Oncol.

[2] Dieci MV, Prat A, Tagliafico E, Pare L, Ficarra G, Bisagni G (2016). Integrated evaluation of PAM50 subtypes and immune modulation of pCR in HER2-positive breast cancer patients treated with chemotherapy and HER2-targeted agents in the CherLOB trial. Ann Oncol.

[3] Ingold Heppner B, Untch M, Denkert C, Pfitzner BM, Lederer B, Schmitt W (2016). Tumor-infiltrating lymphocytes: a predictive and prognostic biomarker in Neoadjuvant-treated HER2-positive breast cancer. Clin Cancer Res.

[4] Loi S, Michiels S, Salgado R, Sirtaine N, Jose V, Fumagalli D (2014). Tumor infiltrating lymphocytes are prognostic in triple negative breast cancer and predictive for trastuzumab benefit in early breast cancer: results from the FinHER trial. Ann Oncol.

[5] Loi S, Sirtaine N, Piette F, Salgado R, Viale G, Van Eenoo F (2013). Prognostic and predictive value of tumor-infiltrating lymphocytes in a phase III randomized adjuvant breast cancer trial in node-positive breast cancer comparing the addition of docetaxel to doxorubicin with doxorubicin-based chemotherapy: BIG 02-98. J Clin Oncol.

[6] Luen SJ, Salgado R, Fox S, Savas P, Eng-Wong J, Clark E (2017). Tumour-infiltrating lymphocytes in advanced HER2-positive breast cancer treated with pertuzumab or placebo in addition to trastuzumab and docetaxel: a retrospective analysis of the CLEOPATRA study. Lancet Oncol.

[7] Salgado R, Denkert C, Campbell C, Savas P, Nuciforo P, Aura C (2015). Tumor-infiltrating lymphocytes and associations with pathological complete response and event-free survival in HER2-positive early-stage breast cancer treated with Lapatinib and Trastuzumab: a secondary analysis of the NeoALTTO trial. JAMA Oncol.

[8] Salgado R, Denkert C, Demaria S, Sirtaine N, Klauschen F, Pruneri G (2015). The evaluation of tumor-infiltrating lymphocytes (TILs) in breast cancer: recommendations by an international TILs working group 2014. Ann Oncol.

[9] Denkert C, Wienert S, Poterie A, Loibl S, Budczies J, Badve S (2016). Standardized evaluation of tumor-infiltrating lymphocytes in breast cancer: results of the ring studies of the international immuno-oncology biomarker working group. Mod Pathol.

[10] Liu S, Duan X, Xu L, Xin L, Cheng Y, Liu Q (2015). Optimal threshold for stromal tumor-infiltrating lymphocytes: its predictive and prognostic value in HER2-positive breast cancer treated with trastuzumab-based neoadjuvant chemotherapy. Breast Cancer Res Treat.

[11] Hanahan D, Weinberg RA (2011). Hallmarks of cancer: the next generation. Cell.

[12] Stack EC, Wang C, Roman KA, Hoyt CC (2014). Multiplexed immunohistochemistry, imaging, and quantitation: a review, with an assessment of Tyramide signal amplification, multispectral imaging and multiplex analysis. Methods.

[13] Levenson RM, Borowsky AD, Angelo M (2015). Immunohistochemistry and mass spectrometry for highly multiplexed cellular molecular imaging. Lab Investig.

[14] Feng Z, Puri S, Moudgil T, Wood W, Hoyt CC, Wang C (2015). Multispectral imaging of formalin-fixed tissue predicts ability to generate tumor-infiltrating lymphocytes from melanoma. J Immunother Cancer.

[15] Angell HK, Gray N, Womack C, Pritchard DI, Wilkinson RW, Cumberbatch M (2013). Digital pattern recognition-based image analysis quantifies immune infiltrates in distinct tissue regions of colorectal cancer and identifies a metastatic phenotype. Br J Cancer.

[16] Degnim AC, Brahmbhatt RD, Radisky DC, Hoskin TL, Stallings-Mann M, Laudenschlager M (2014). Immune cell quantitation in normal breast tissue lobules with and without lobulitis. Breast Cancer Res Treat.

[17] Johansson AC, Visse E, Widegren B, Sjogren HO, Siesjo P (2001). Computerized image analysis as a tool to quantify infiltrating leukocytes: a comparison between high- and low-magnification images. J Histochem Cytochem.

[18] Tumeh PC, Harview CL, Yearley JH, Shintaku IP, Taylor EJ, Robert L (2014). PD-1 blockade induces responses by inhibiting adaptive immune resistance. Nature.

[19] Lopez C, Callau C, Bosch R, Korzynska A, Jaen J, Garcia-Rojo M (2014). Development of automated quantification methodologies of immunohistochemical markers to determine patterns of immune response in breast cancer: a retrospective cohort study. BMJ Open.

[20] Vasaturo A, Di Blasio S, Verweij D, Blokx WA, van Krieken JH, de Vries IJ (2016). Multispectral imaging for highly accurate analysis of Tumor Infiltrating Lymphocytes in primary melanoma. Histopathology.

[21] Oguejiofor K, Hall J, Slater C, Betts G, Hall G, Slevin N (2015). Stromal infiltration of CD8 T cells is associated with improved clinical outcome in HPV-positive oropharyngeal squamous carcinoma. Br J Cancer.

[22] Garnelo M, Tan A, Her Z (2015). Yeong J.

[23] Wolff AC, Hammond ME, Hicks DG, Dowsett M, McShane LM, Allison KH (2013). Recommendations for human epidermal growth factor receptor 2 testing in breast cancer: American Society of Clinical Oncology/College of American Pathologists clinical practice guideline update. J Clin Oncol.

[24] Ruifrok AC, Johnston DA (2001). Quantification of histochemical staining by color deconvolution. Anal Quant Cytol Histol.

[25] Keshava N, Mustard JF (2002). Spectral unmixing. IEEE Signal Process Mag.

[26] Gertych A, Mohan S, Maclary S, Mohanty S, Wawrowsky K, Mirocha J (2014). Effects of tissue decalcification on the quantification of breast cancer biomarkers by digital image analysis. Diagn Pathol.

[27] Zack GW, Rogers WE, Latt SA (1977). Automatic measurement of sister chromatid exchange frequency. J Histochem Cytochem.

[28] Young IT (1983). Image analysis and mathematical morphology, by J. Serra. Academic press, London, 1982, xviii + 610 p. $90.00. Cytometry.

[29] Nomizu K, Sasaki S. Affine Differential Geometry (New ed.). Melbourne: Cambridge University Press; 1994.

[30] Gertych A, Ing N, Ma Z, Fuchs TJ, Salman S, Mohanty S (2015). Machine learning approaches to analyze histological images of tissues from radical prostatectomies. Comput Med Imaging Graph.

[31] Pozner-Moulis S, Cregger M, Camp RL, Rimm DL (2007). Antibody validation by quantitative analysis of protein expression using expression of met in breast cancer as a model. Lab Investig.

[32] Huang F, Ma Z, Pollan S, Yuan X, Swartwood S, Gertych A (2016). Quantitative imaging for development of companion diagnostics to drugs targeting HGF/MET. J Pathol Clin Res.

[33] Ginter PS, Varma S, Liu YF, Shin SJ (2015). The minimal carcinoma triple stain is superior to commercially available multiplex immunohistochemical stains: breast triple stain and LC/DC breast cocktail. Am J Clin Pathol.

[34] Brown JR, Wimberly H, Lannin DR, Nixon C, Rimm DL, Bossuyt V (2014). Multiplexed quantitative analysis of CD3, CD8, and CD20 predicts response to neoadjuvant chemotherapy in breast cancer. Clin Cancer Res.

[35] Di Palma S, Bodenmiller B (2015). Unraveling cell populations in tumors by single-cell mass cytometry. Curr Opin Biotechnol.

