# Supplementary figures and images for: Data integration from pathology slides for quantitative imaging of multiple cell types within the tumor immune cell infiltrate
Source: Diagn Pathol. 2017 Sep 18;12:69. doi: 10.1186/s13000-017-0658-8 (PMC5604347; doi:10.1186/s13000-017-0658-8)

## Slide 1
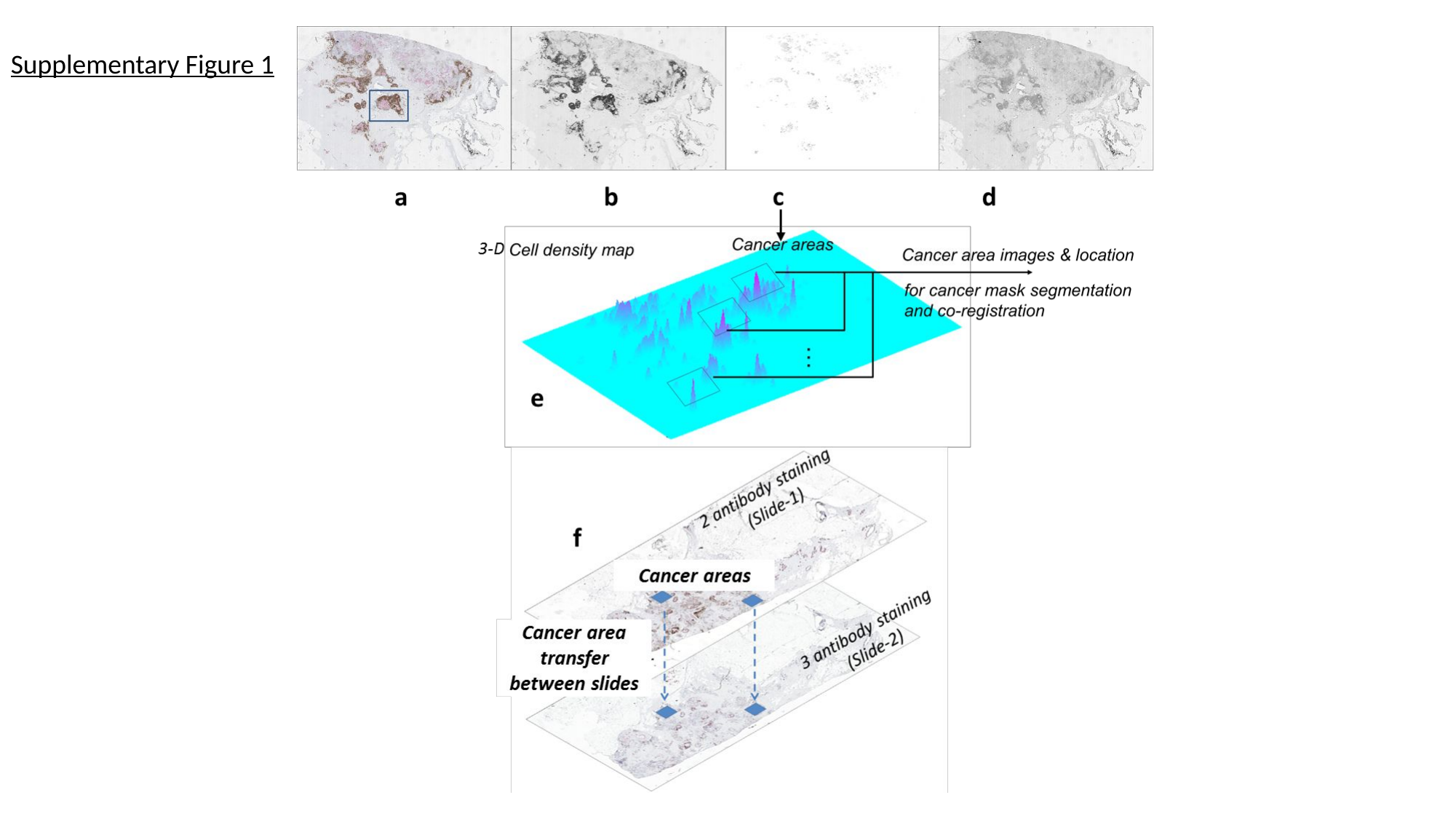

Supplementary Figure 1

## Slide 2
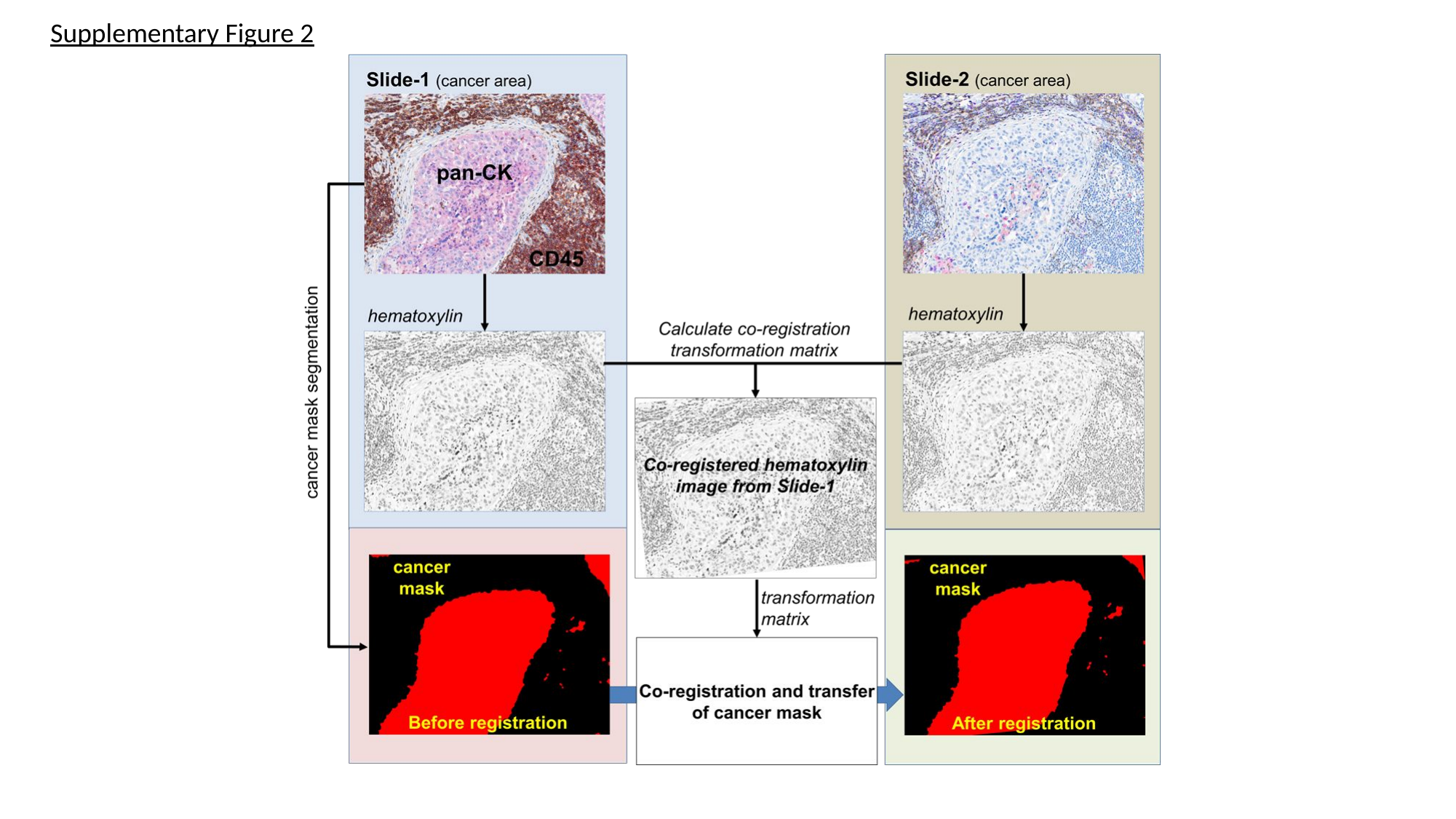

Supplementary Figure 2

Supplement: Additional file 1:Fig. S1 — Visualization of staining components used to generate a 3-D cell density map of cancer areas. (a) A whole-slide multicolor RGB bright-field image of breast cancer tissue stained for CD45 (brown), Pan-CK (red) and a hematoxylin nuclear counter-stain (blue). (b-d) The whole-slide multicolor RGB image was color-deconvoluted to separate the staining components: (b) CD45+ image, (c) Pan-CK+ image, and (d) hematoxylin image. (e) A 3-D cell density map of Pan-CK-positive cells (which demarcates areas of epithelial cells) was derived from the Pan-CK component image. Areas of benign glands and in-situ ductal carcinoma were visually excluded by a pathologist. A threshold applied to the map lead to the identification of cancer areas which were processed to segment out a cancer mask. (f) The locations of the cancer mask were transferred to a tissue image with no Pan-CK staining to enable the enumeration of immune cells in IHC stained slides. Fig. S2 Transfer of cancer mask from slide-1 to slide-2. The cancer mask is generated in slide-1 based on Pan-CK staining. Hematoxylin-stained nuclei in slide-1 are identified and co-registered with corresponding, hematoxylin-stained nuclei in slide-2. A transformation matrix is established based on co-registered nuclei to transform the cancer mask in slide-1 for alignment with slide-2 in the whole ROI. After co-registration the cancer mask is transferred from slide-1 to slide-2. (PPTX 1673 kb) [file 13000_2017_658_MOESM1_ESM.pptx]
